# Electroacupuncture Alleviates Diabetic Peripheral Neuropathy by Regulating Glycolipid-Related GLO/AGEs/RAGE Axis

**DOI:** 10.3389/fendo.2021.655591

**Published:** 2021-07-06

**Authors:** Xuan Wang, Qian Li, Xu Han, Meirong Gong, Zhi Yu, Bin Xu

**Affiliations:** Key Laboratory of Acupuncture and Medicine Research of Ministry of Education, Nanjing University of Chinese Medicine, Nanjing, China

**Keywords:** diabetes, electroacupuncture, peripheral neuropathy, glyoxalase system, advanced glycation end products, neuropathic pain

## Abstract

Diabetic peripheral neuropathy (DPN) is one of the most common complications of diabetes mellitus (DM) and affects over one-third of all patients. Neuropathic pain and nerve dysfunction induced by DM is related to the increase of advanced glycation end products (AGEs) produced by reactive dicarbonyl compounds in a hyperglycemia environment. AGEs induce the expression of pro-inflammatory cytokines *via* the main receptor (RAGE), which has been documented to play a crucial role in the pathogenesis of diabetic peripheral neuropathy. Electroacupuncture (EA) has been reported to have a positive effect on paralgesia caused by various diseases, but the mechanism is unclear. In this study, we used high-fat-fed low-dose streptozotocin-induced rats as a model of type 2 diabetes (T2DM). Persistent metabolic disorder led to mechanical and thermal hyperalgesia, as well as intraepidermal nerve fiber density reduction and nerve demyelination. EA improved neurological hyperalgesia, decreased the pro-inflammatory cytokines, reduced the generation of AGEs and RAGE, and regulated the glyoxalase system in the EA group. Taken together, our study suggested that EA plays a role in the treatment of T2DM-induced DPN, and is probably related to the regulation of metabolism and the secondary influence on the GLO/AGE/RAGE axis.

## Introduction

Diabetes mellitus (DM) is a metabolic disorder characterized by hyperglycemia, hyperlipidemia, and glycosuria. It has become epidemic worldwide and has one of the highest incidence rates of chronic diseases. The International Diabetes Federation recently indicated that more than 463 million people have diabetes and the number of cases was expected to increase to 700 million by 2045 (1). Type 2 diabetes (T2DM) is recognized as the most common. The increasing prevalence of diabetes and its secondary complications have created a huge economic burden around the world (2), and among the complications, diabetic peripheral neuropathy (DPN) especially type 2 is the most common and troublesome. Statistics from the Center for Disease Control and Prevention showed that over half of diabetic patients will develop DPN and over one-third will develop neuropathic pain during the process of DM (3, 4). The complexity and increased prevalence of DPN have inflicted a burden on human health.

Inflammation plays a critical role in painful DPN (5). Pro-inflammatory cytokines such as TNF-α, interleukin 1 (IL 1), and IL 6 have been reported to be important in peripheral sensitization. In the development of DM, metabolism disorder resulting from hyperglycemia contributes to inflammatory signaling mechanisms, leading to the energy stress of mitochondria and axons, and eventually causes nerve injury (6). Compounds that inhibit inflammatory response have been confirmed to be effective in the treatment of DPN (7, 8), indicating that reducing inflammation and blocking the cascade is an efficient therapy.

Advanced glycation end products (AGEs) produced by non-enzymatic reactions contribute to intra- and extracellular protein cross-link and essential protein modification. AGEs deposit in almost every part of nerve tissues and the deposition is related to the density reduction of myelinated nerve fibers (9). Besides, AGEs can result in nerve dysfunction by interacting with cell surface receptors essentially the receptor for AGEs (RAGE), activating the downstream signal cascade, causing a persistent inflammatory reaction and neurological damage and promoting the development of diabetic neuropathy (10), which is one of the main types of pathogenesis of DPN.

Methylglyoxal (MG) is the main precursor of AGEs. In the continuous hyperglycemia environment, the increase of the MG level leads to the accumulation of AGEs. As the main rate-limiting enzyme of the glyoxalase system, glyoxalase-1 (GLO1) detoxifies MG, with glutathione (GSH) as a cofactor. The expression and function of GLO1 decrease under hyperglycemia while RAGE increases, which aggravates the deposition and signal transduction of AGEs. Enhancing GLO1 expression will prevent MG-induced formation of AGEs, decrease the downstream inflammatory signal cascade, and reduce the impairment of the nerve system, which indicates its potential importance in peripheral nerve system protection. Therefore, targeting the activation of GLO1 and the formation of AGEs will be more effective in treatment.

Multiple studies showed the anti-inflammatory effect of electroacupuncture (EA) on various forms of organ dysfunction including many diabetic complications (11–13). Electrical nerve stimulation has been confirmed to reduce pain in diabetes by a large magnitude (14). It has been endorsed by the American Pain Society and the National Center for Complementary and Alternative Medicine because of its effective therapy and is used by millions of people to reduce pain and block inflammation (15). Previous research reported the therapeutic effect of EA treatment in the relief of hyperalgesia caused by a range of reasons including diabetes-associated hyperalgesia and its effect on the reduction of the level of AGEs and RAGE (16–19). However, it is unclear whether this reduction is relevant to hyperalgesia relief and the particular mechanism. ST25 (Tianshu) has been used in clinical trails widely (20, 21). It has been reported that high-intensity ST25-EA stimulation (1.0-3.0 mA) modulated systemic inflammation by activating distinct sympathetic pathways (22). ST25-EA stimulation was confirmed to regulate the activity of glucose-inhibited neurons and improve the disorder of lipid metabolism (23). Our previous research showed that the positive effect on obesity mediated by ST25-EA stimulation was probably associated with the promotion of mitochondrial biogenesis and the regulation of immunologic balance (24, 25). Additionally, ST25 was confirmed to relieve various forms of pain including visceral hyperalgesia and cancer pain (26–28).

In this research, we used high-fat-fed/low-dose streptozotocin (HFD-STZ)-induced rats as T2DM models to recapitulate the metabolic characteristics in T2DM-induced DPN. Low-dose streptozotocin (35mg/kg) mildly inhibits beta-cell function, and combined with insulin resistance caused by the HFD, results in hyperglycemia (29). It is different from high-dose STZ-induced diabetes, in which the hyperglycemia results from beta-cell dysfunction-induced insulin deficiency (30, 31). Herein, we observed that EA alleviated hyperalgesia and metabolic disorder in model rats, and inferred that the levels of inflammation and the GLO/AGE/RAGE axis might be influenced by that. It may provide further understanding of EA treatment in DPN.

## Materials and Methods

### Animals and Groups

Eight-week-old Sprague-Dawley male rats weighing approximately 200-220 g, which were purchased from the Model Animal Research Center of Nanjing Medical University, were housed in a controlled temperature room (20-22°C) with relative humidity of 40%-60%, a 12-h/12-h light/dark cycle, and ad libitum access to food and water. All of the rats’ experiments were performed according to the “Guide for the Care and Use of Laboratory Animals” published by the National Institutes of Health and with the protocols approved by the Institutional Animal Care and Use Committee of Nanjing University of Chinese Medicine (Animal license number: SCXK_2019-0002). The rats were divided into three groups (control, model, and EA) and were placed on a basic diet in the first two weeks. Two weeks later, one of these groups was designated to be the control group and kept on a basic diet, while the other two groups were placed on a high-fat-fed diet (30% fat) and designated as type 2 diabetes groups. Another two weeks later, hyperglycemia was induced by intraperitoneal injection of STZ (35mg/kg, 0.1 M citric acid buffer, pH 4.5) in the two high-fat-fed groups, and the control group was treated with vehicle. One week after STZ injection, the rats in the high-fat-fed groups with blood glucose >16.7mmol/L were used in experiments.

### EA Stimulation

The rats in the EA group were anesthetized by inhaled isoflurane (4-5% for induction and 1-2% for maintenance) and placed on a heating pad to maintain body temperature. EA was performed with a continuous-wave stimulation for 20 min, with an alternating frequency of 2/15 Hz and a current of 2 mA (23, 25). A pair of non-insulated steel acupuncture needles (0.18 mm in diameter, 10 mm in length) were inserted at a depth of 3 mm on ST25 (Tianshu, locating 5 mm laterally to the intersection between the upper 2/3 and the lower 1/3 in the line joining the xiphoid process and the upper border of the pubic symphysis), and the needles were connected to the output terminals of the EA instrument (LH402A; Beijing Huawei Technologies Co. Ltd). This treatment was performed six times a week and lasted for 5 weeks. To minimize the extra stimulus and stress, EA stimulation was carried out with an extremely gentle operation on the rats. The gas anesthesia was given to rats in the model group at the same time without performing EA.

### Behavioral Test

The behavioral test was conducted every week after the STZ injection to check if the rats were in hyperalgesia. All of the behavioral measurements were carried out when the rats were awake and unrestrained.

#### Hind Paw Withdrawal Threshold

Von Frey measurements were done after the rats were placed on the wire for half an hour and had adapted to the environmental divorce to check mechanism sensitivity. A Von Frey filament was forced against the hind paw and rose at a uniform speed until the rats were lifting their hind feet. The force in grams exerted by wire on the hind paw increased with time. When the rats withdrew their hind paw, the force stopped increasing, and the corresponding force was regarded as the withdrawal threshold and was calculated. Each rat was measured five times, alternately on the left and right hind paw, with an interval of 5 min (32).

#### Hind Paw Withdrawal Latency

Hind paw withdrawal latency was measured with an analgesia meter. Rats were placed on a warm plate (30°C) for half an hour to adapt to the environmental temperature. The light source was maneuvered under the hind paw, starting at 30°C and ending at 55°C to avoid scalding the skin. The paw withdrawal time was recorded to measure the sensitivity of heat. The time was limited to 30 s. Each rat was measured four times, alternately on the left and right hind paw, with an interval of 5 min (33).

### Nerve Conduction Velocity

Motor nerve conduction velocity (MNCV) and sensory nerve conduction velocity (SNCV) were recorded in the posterior-sciatica tibial conduction system using PowerLab 8/35 (AD Instruments, Australia). The rats were anesthetized by intraperitoneal injection of urethane (1200mg/kg) and body temperature was maintained at 37°C during the measurement. The sciatic nerve near the sciatic notch and the tibial nerve near the Achilles tendon were stimulated with a single stimulus of 3 V. The M-wave (used for MNCV calculation) and H-wave (used for SNCV calculation) reflexes were recorded by the receipt electrodes placed on the interosseous foot muscle (34–36).

### Microcirculatory Blood Perfusion

The microcirculatory blood perfusion units of the dorsal hind paw were measured by a Laser Doppler (PeriFlux5000, Perimed, Sweden). The rats were anesthetized with isoflurane, 4-5% for induction and 1-2% for maintenance (37), and were then placed on a heating pad to keep their temperature around 37°C. After removing the hair on the measuring area, the probe (Probe 408) was vertically fixed on the skin with double-sided adhesive tape (PF105-1), and attention was paid to avoid the blood vessels on the surface. When the baseline was stable, the perfusion units (PU) of each rat were recorded three times, 3 min each time with an interval of 5 min. The value of the PU was defined as the product of the concentration of moving blood cells and the average movement rate of blood cells.

### Biochemical Analyses

Levels of blood glucose and body weight were measured every week and the measurements were carried out at the same time. Blood glucose was measured by an ACCU-CHEK Performa (Roche Diabetes Care GmbH). The levels of insulin, high-density lipoprotein, low-density lipoprotein, glycosylated serum protein, triglyceride, non-esterified fatty acid, total cholesterol in serum, and glutathione in hind paw skin were measured with a related assay kit (Nanjing Jiancheng Bioengineering Institute, Nanjing) at the end of the experiment.

### ELISA

Standard or samples (50 μL) with enzyme conjugate (100 μL) (Nanjing Jiancheng Bioengineering Institute, Nanjing) were added to the appropriate wells in the 96-well polystyrene microplates, covered with an adhesive strip, and incubated for 60 min at 37°C. The incubation mixture, aspirate and wash solution (1X, 350 μL), was filled in each well and this procedure was repeated five times. After the final wash, the blot dried in the plate and different substrates were added (50 μL) and incubated for 15 min at 37°C, protected from light. Finally, the stop solution (50 μL) was added to each well and the optical density was read at 450 nm using a microtiter plate reader within 15 min.

### Hematoxylin and Eosin (H&E) Staining

After being carefully isolated, the sciatic nerves were fixed in 4% paraformaldehyde and embedded in paraffin wax. Then 2 μm semithin sections were taken with a rotary slicer (Leica, Germany) and mounted on the slides. Hematoxylin and eosin (H&E) staining was performed under a light microscope (Olympus, Japan) to observe for pathological changes (38).

### Immunohistochemistry Staining (IHC)

Hind paw skin was fixed with 4% paraformaldehyde and sectioned into 20 μm pieces. The sections were treated with 3% hydrogen peroxide to block the activity of endogenous peroxidase and incubated with 5% goat serum at 37°C for 30 min. After the reaction with the primary antibody PGP9.5 (1:200, Santa Cruz), secondary antibody, SABC, and chromogenic agent were dripped onto the sections in turn, and then they were re-stained with hematoxylin. Finally, sections were covered with neutral gum and examined under the light microscope (39).

### Western Blotting Analysis

Hind paw skin and sciatic nerves were isolated and washed in normal saline. A total of 100 mg of tissue was placed in 300 μL of lysis buffer, which consists of protease inhibitor and RIPA (Thermo Scientific), was homogenized and centrifuged at 12000 r/min for 30 min. After that, the protein concentrations were measured with a BCA Protein Assay Kit (Thermo Scientific). Then, 15 μg of protein from each sample was separated on SDS-PAGE and transferred to PVDF membranes. Next, the membranes were blocked in 5% BSA for 1.5 h and incubated with primary antibodies PGP9.5 (1:200, Santa Cruz), AGE (1:500, Abcam), RAGE (1:1000, Abcam), and GLO1 (1:200, Santa Cruz) overnight at 4°C. After incubating with the corresponding secondary antibodies (1:5000, Abways) at room temperature for 1 h, the membranes were analyzed by enhanced chemiluminescence detection.

### Statistical Analysis

Data analysis was performed by SPSS 24.0 (IBM Corp., Armonk, NY, USA) and GraphPad Prism 8.0 (GraphPad Inc., La Holla, CA, USA) and presented as mean ± SEM. Two groups were compared using two-tailed Student’s t-tests and more than two groups were compared using one-way ANOVA. The images of immunostaining with H&E were analyzed by ImageJ. * means compared to control, # means compared to model. A value of p<0.05 was defined as significant.

## Results

### Effect of EA on Representative Symptoms of T2DM Replicated in HFD-STZ Rat Models

**Figure 1:**
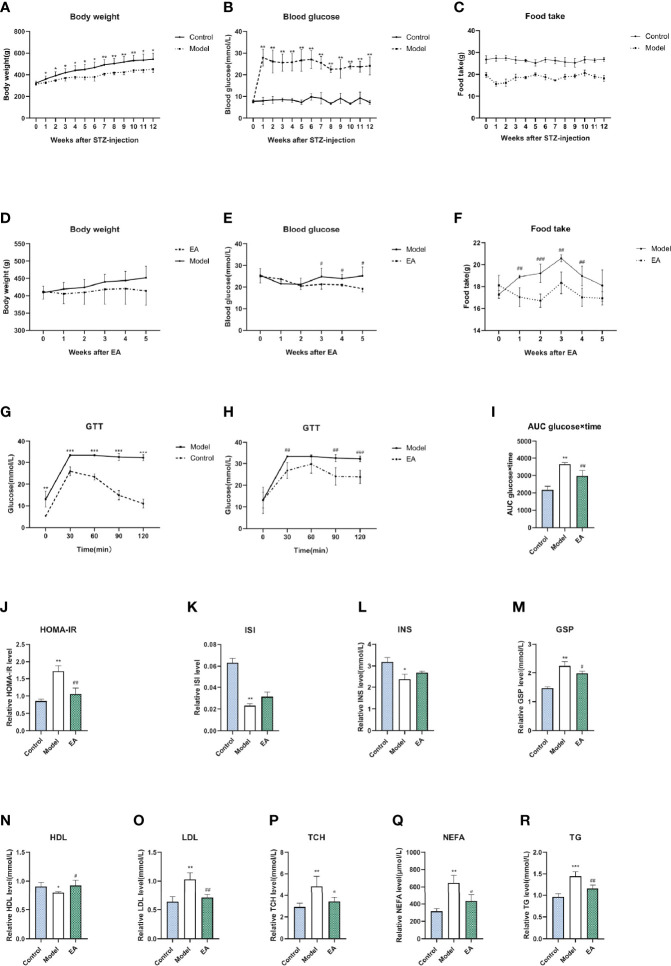
Metabolism index of HFD-STZ rats in different groups. Levels of body weight **(A)**, blood glucose **(B)**, food intake **(C)**, and glucose tolerance test (GTT) **(G)** in the control group and model group over 12 weeks (n = 6, *p < 0.05, **p < 0.01, ***p < 0.001). The significance was not marked in the figure since the food was not the same. Compared to the model group, EA treatment decreased body weight **(D)**, blood glucose **(E)**, food intake **(F)**, as well as hyperglycemia in GTT **(H)** and related AUC levels **(I)** significantly (n = 6, ^#^P < 0.05, ^##^P < 0.01, ^###^P < 0.001). Levels of homeostasis model assessment for insulin resistance **(J)**, insulin sensitivity index level **(K)**, fasting insulin level **(L)**, glycosylated serum protein level **(M)**, high density lipoprotein level **(N)**, low density lipoprotein level **(O)**, total cholesterol level **(P)**, non-esterified fatty acid **(Q)**, and triglycerides level **(R)** in serum of rats after saline, HFD-STZ-induced, and EA treatment (n = 4, *p < 0.05, **p < 0.01, ***p < 0.001, ^#^p < 0.05, ^##^p < 0.01).

We measured blood glucose levels and body weight to evaluate the successful induction of models. Compared with the control group, STZ injection resulted in a significant decrease in body weight (**Figure 1A**). Significant disorder of glucose metabolism including hyperglycemia (blood glucose >16.7mmol/L) (**Figure 1B**), insulin resistance (**Figures 1J–L**), glucose tolerance reduction (**Figures 1G–I**), and GSP level (**Figure 1M**) as well as lipid metabolism such as levels of HDL, LDL, TCH, NEFA, and TG (**Figures 1N–R**) were observed in the model group, which were consistent with clinical diabetes. EA treatment decreased blood glucose, body weight, and food intake levels significantly compared to the model group (**Figures 1D–F**). Taken together, hyperglycemia and dyslipidemia were improved in the EA group, suggesting the therapeutic effect of EA in T2DM.

### Effect of EA on the Behavioral Test, Nerve Conduction Velocity, and Microcirculatory Blood Perfusion of STZ-HFD-Induced Rats

**Figure 2:**
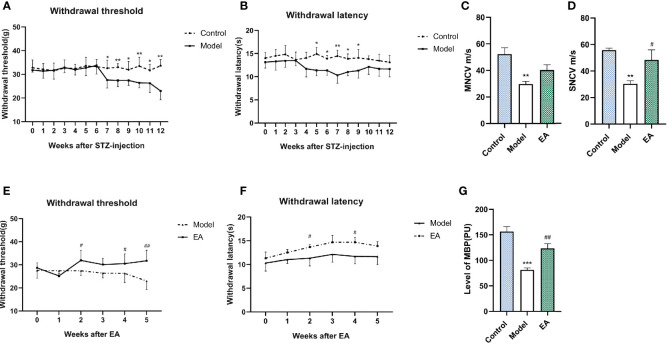
Effect of EA on withdrawal threshold, withdrawal latency, nerve conduction velocity, and microcirculatory blood perfusion. Withdrawal threshold **(A)** and latency **(B)** were evaluated weekly after STZ injection and improved significantly after EA treatment compared to the control group **(E, F)** (n = 6, *p < 0.05, **p < 0.01, ^#^p < 0.05, ^##^p < 0.01). Motor nerve conduction (MNCV) **(C)** and sensory conduction velocity (SNCV) **(D)** in the control group, the model group, and the EA group (n=4, **p < 0.01, ^#^p < 0.05). MBP **(G)** in the control group, the model group, and the EA group (n = 6, ***p < 0.001, ^##^p < 0.01).

After STZ injection, hyperalgesia was observed in the model group and lasted for the whole experimental period (**Figures 2A, B**). Motor nerve conduction velocity (MNCV) and sensory conduction velocity (SNCV) were reduced in the model group (**Figures 2C, D**). MBF (microcirculatory blood perfusion) of the hind paw was significantly reduced as well (**Figure 2G**). EA treatment improved mechanical hyperalgesia and thermal latency significantly (**Figures 2E, F**). The MNCV, SNCV (**Figures 2C, D**), and MBF (**Figure 2G**) were observed to increase after 5 weeks of EA treatment.

### Effect of EA on Intraepidermal Nerve Fiber Density and Histopathology of Sciatic Nerve in STZ-HFD-Induced Rats

**Figure 3:**
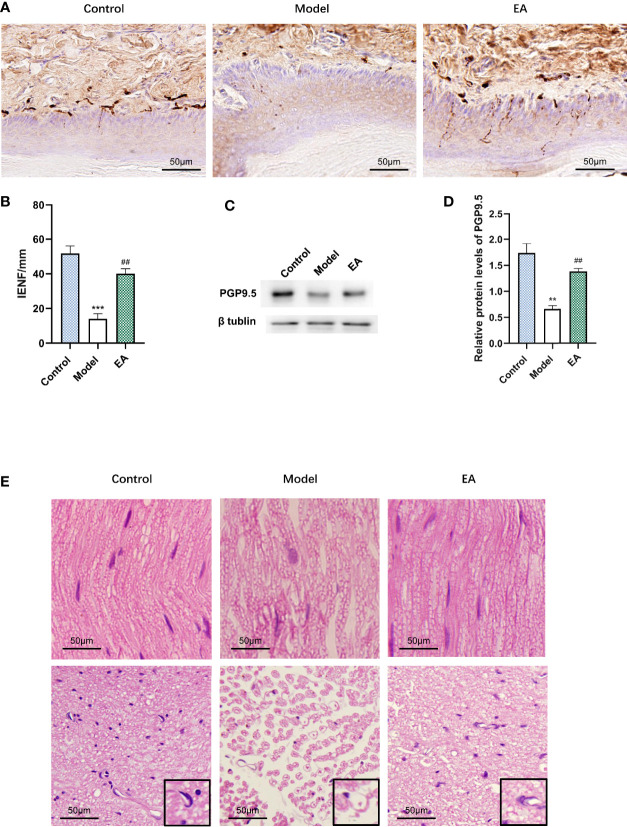
Effect of EA on IENF, the expression of PGP9.5, and histopathology of the sciatic nerve. **(A)** Immunohistochemistry staining for PGP9.5 in hind paw skin of rats and **(B)** measurement of IENF (n = 3, ***p < 0.001, ^##^p < 0.01. Scale bar, 50 μm). **(C)** Representative Western blot analysis of PGP9.5 staining in footpad skin and **(D)** relative protein levels (n = 3, **p < 0.01, ^##^p < 0.01). **(E)** Morphological examination of paraffin-sectioned sciatic nerves performed at 12 weeks after STZ-HFD induction. A single nerve and its myelin sheath were observed in a high-power microscope (×400). (n = 3, scale bar, 50 μm).

Immunohistochemistry staining was performed for PGP9.5 to evaluate the effect of EA on intraepidermal nerve fiber density (IENF). It was observed that IENF in the model group was less than that in the control one, and EA treatment led to a significant increase (**Figures 3A, B**). The relative protein level of PGP9.5 in hind paw skin was consistent with this result (**Figures 3C, D**). To examine the histopathology changes induced by diabetes, we performed H&E staining of different sections of the sciatic nerve. Nerves of the model group were shown to be disordered and the myelinated fibers were smaller than in the control group (**Figure 3E**). After EA treatment, the structure of the sciatic nerve was so improved that the morphology of the myelin sheath was more complete compared to the model group (**Figure 3E**).

### Effect of EA on Inflammatory State of the Footpad Skin and the Sciatic Nerve in HFD-STZ Rats

**Figure 4:**
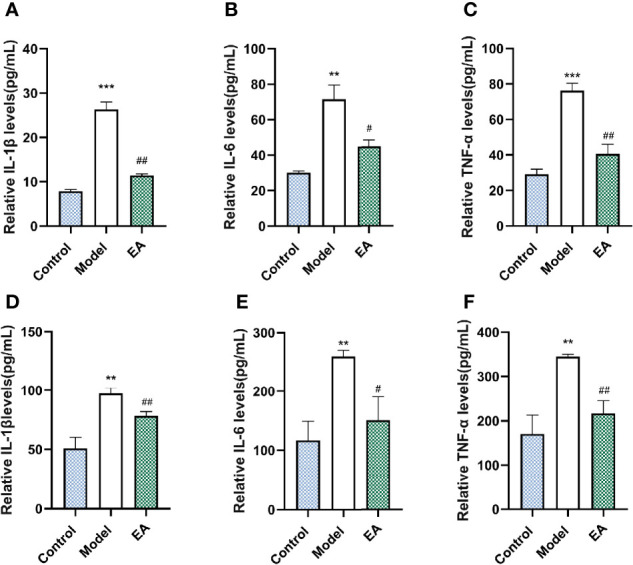
Effect of EA on the expression of IL 1β, IL 6, and TNF-α in the footpad skin and the sciatic nerve. Relative levels of IL 1β **(A)**, IL 6 **(B)**, and TNF-α **(C)** of footpad skin in the control, model, and EA group (n = 4, **p < 0.01, ***p < 0.001, ^#^p < 0.05, ^##^p < 0.01). Relative levels of IL 1β **(D)**, IL 6 **(E)**, and TNF-α **(F)** of footpad skin in the control, model, and EA group (n = 3, **p<0.01, ^#^p < 0.05, ^##^p < 0.01).

To examine the effect of EA on the inflammatory state, we measured pro-inflammatory cytokines in the HFD-STZ rats’ footpad skin and sciatic nerve, which were reported to be closely associated with diabetic peripheral neuropathy (5). Levels of IL 1β, IL 6, and TNF-α in the model groups were significantly higher than those in the control group both in the footpad skin (**Figures 4A–C**) and the sciatic nerve (**Figures 4D–F**). EA treatment alleviated the levels of these three pro-inflammatory cytokines, suggested its anti-inflammatory effects (**Figures 4A–F**).

### Effect of EA on the Expression of AGEs and RAGE in the Footpad Skin and the Sciatic Nerve

**Figure 5:**
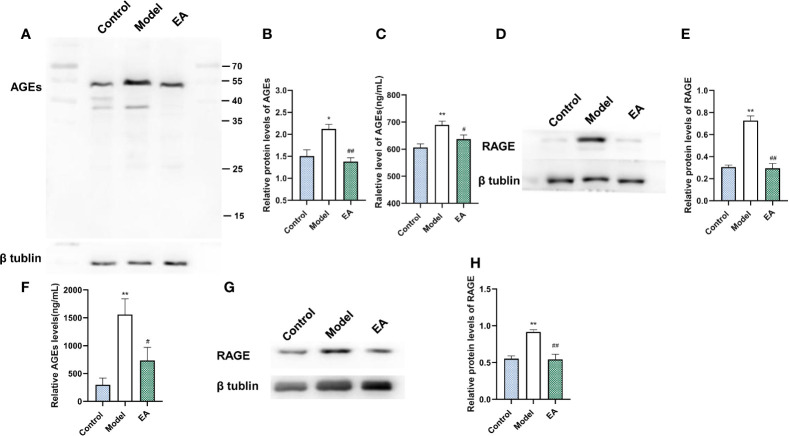
Effect of EA on the expression of AGEs and RAGE in footpad skin and sciatic nerve. **(A)** Representative Western blot analysis of AGEs staining and **(B)** relative protein levels in footpad skin (n = 3, *p < 0.05, ^##^p < 0.01). **(C)** Representative ELISA analysis of AGEs in footpad skin (n = 3, **p < 0.01, ^#^p < 0.05). **(D)** Representative Western blot analysis of RAGE staining and **(E)** relative protein levels in footpad skin (n = 3, **p < 0.01, ^##^p < 0.01). **(F)** Representative ELISA analysis of AGEs in the sciatic nerve (n = 3, **p < 0.01, ^#^p < 0.05). **(G)** Representative Western blot analyses of RAGE staining and **(H)** relative protein levels in the sciatic nerve (n = 3, **p < 0.01, ^##^p < 0.01).

It is reported that AGE binds to its receptor RAGE, promotes the expression of inflammatory signals, and further damages nerve fibers. To investigate the effect of EA on the expression of AGEs and their main receptor (RAGE), we measured the expression of AGEs and RAGE in both the footpad skin and the sciatic nerve. The levels of AGEs and RAGE of footpad skin in the model group were significantly increased compared with the control group (**Figures 5A–E**) and EA treatment reduced their expression (**Figures 5A–E**). Similar results were also found in sciatic nerves by Western blot and ELISA (**Figures 5F–H**).

### Effect of EA on the Glyoxalase Pathway

**Figure 6:**
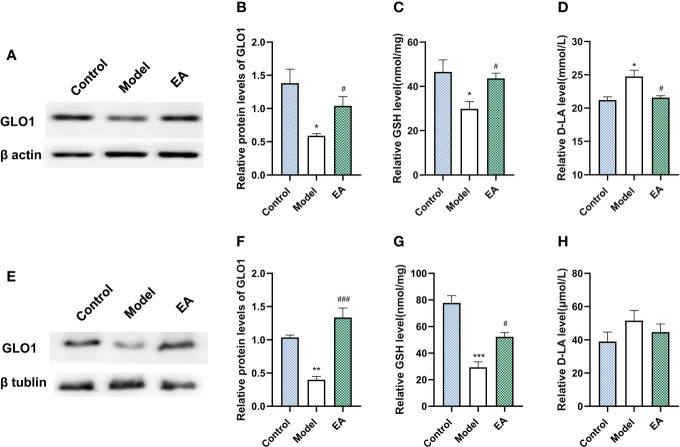
Effect of EA on the expression of GLO1, GSH, and D-lactate in footpad skin and sciatic nerve. **(A)** Representative Western blot analysis of GLO1 staining and **(B)** relative protein levels in footpad skin (n=3, *p < 0.05, ^#^p < 0.05). **(C)** Representative ELISA analysis of GSH and **(D)** D-lactate in footpad skin (n=4, *p < 0.05, ^#^p < 0.05). **(E)** Representative Western blot analysis of GLO1 staining and **(F)** relative protein levels in sciatic nerve (n=3, **p < 0.01, ^###^p < 0.001). **(G)** Representative ELISA analysis of GSH and **(H)** D-lactate in sciatic nerve (n=4, ***p < 0.001, ^#^p < 0.05).

GLO1 plays a role in the detoxification of dicarbonyl compounds, with GSH as a cofactor and D-lactate as a metabolite, and is the main rate-limiting enzyme of the glyoxalase system. Diabetes significantly decreased the expression of GLO1 and GSH (**Figures 6A–C**) in footpad skin compared with the control group. EA significantly increased the levels of GLO1 and GSH (**Figures 6A–C**, p<0.05). Moreover, EA decreased the levels of D-lactate (**Figure 6D**, p<0.05) which increased in the model group. Similar results were observed in sciatic nerves (**Figures 6E–H**).

## Discussion

Acupuncture has been proven to play a role in regulating metabolism and relieving pain and is wildly used in diabetes. However, few studies target the relationship and the underlying mechanism between these two effects. In this research, a HFD-STZ-induced model was recruited for its characteristics of DPN and metabolism disorder. T2DM is one of the most prevalent diseases in the world (40, 41). HFD-STZ-induced models are confirmed to have a more similar phenotype, pathogenesis, and other human-like conditions than genetic and chemical models, and are wildly used in related research (42–45), especially low-dose STZ (35 mg/kg) injection, which has been further proved to offer metabolic syndrome replication and relatively stable elevated glucose concentrations following T2DM. Different from high-dose STZ injection, which completely induces beta-cell impairment, low-dose STZ injection modestly injures beta-cells, in which the serum insulin is maintained at a medium level (**Figure 1L**), and T2DM is stable without an insulin intake requirement (46), that is suitable for studies on diabetic complications including neuropathy (29). A HFD dependably induces a model of other human conditions and has also been utilized for chronic inflammation, and that plays a role in T2DM development (47). In this study, hyperalgesia as well as hyperglycemia, dyslipidemia, and insulin resistance were observed in HFD-STZ-induced rats (**Figure 1**), which is consistent with the clinical characteristics of neuropathy induced in T2DM and suggests the successful conduction of models.

Acupuncture has been used to manage various forms of pain including diabetic pain (48). DPN develops as a result of aberrant myelination, and demyelination is a key mechanism of plasticity in neuropathic pain (49). Mechanical and thermal hyperalgesia (**Figures 2A, B**), nerve conduction velocity decrease (**Figures 2C, D**), as well as aggravation of nerve injury were observed in the model group (**Figures 2A–D, G**). Nerve conduction velocity (NCV) is one of the main diagnostic indicators of DPN and is always used to assess nerve function (50). In this research, it was observed that MNCV and SNCV decreased significantly after T2DM induction. Both animal and human research confirmed that EA promotes preferential re-innervation of both motor and sensory neurons (51–53). EA mediates myelin sheath recovery and axonal regeneration partly through the promotion of axoplasmic mitochondrial proliferation (54). EA treatment also increases the graft neurotrophin and enhances remyelination and functional recovery (55). H&E staining showed that EA treatment partly protects the nerve from demyelination (**Figure 3**). The sparse and disordered sciatic nerve fiber arrangement and the enlarged myelin lamina gap were improved after EA treatment (**Figure 3E**). Hyperglycemia-induced damage to the microvascular system that supplies nerve fibers leads to a significant decrease in microcirculatory blood perfusion and thus injury to the myelin sheath (56).

Metabolic disorder drives the development of diabetic complications including peripheral neuropathy, which is a manifestation of neurological dysfunction and affects up to 60% of T2DM patients (1, 57). A tremendous amount of research suggests that AGEs play a pathogenic role in DPN, whether it is a direct neurotoxic effect or indirect mediating inflammatory injury. AGEs accumulate over axons and myelin sheaths and modify the structural proteins (58), which may cause myelinated fiber reduction (9), nerve dysfunction, and neurotrophic support impairment (59). Hyperglycemia and hyperlipidemia can induce oxidative stress and increase AGEs in different ways. EA has been increasingly used for metabolism-related diseases. EA excites somatic afferent fibers, influences sympathetic nerve activity, increases the secretion of endogenous beta-endorphin, and ameliorates insulin sensitivity (60, 61). The effect of blood glucose improvement was confirmed in both clinical and experimental studies (62, 63). Moreover, it is reported that EA decreases the levels of NPY in the hypothalamus, reduces food intake, and thus improves lipid metabolism (64). In this study, we observed that EA significantly reduced the levels of hyperglycemia (**Figure 2D**), hyperlipidemia (**Figure 2E**), and insulin resistance (**Figure 1J**) in HFD-STZ-induced rats and the metabolism recovery may decrease the formation of AGEs.

Furthermore, the interactions of AGEs and the receptor have been proven to be associated with the development of diabetic neuropathic pain (65). AGEs bind to cell surface receptors like the receptors of AGEs (RAGE), and alters a series of signaling cascades (66, 67), which leads to the increase of neuroinflammation and degeneration (68). Many types of research have confirmed the anti-inflammatory effect mediated by EA. It is reported that EA increases the secretion of endogenous beta-endorphin, suppresses the transduction of pain, and reduces neuroinflammation through the activation of sympathetic nerve fibers in the cholinergic anti‐inflammatory pathway (69–72). We measured the level of RAGE and inflammatory cytokines and observed the decrease of the expression of RAGE (**Figure 4**) and related inflammatory cytokines including IL 1β, IL 6, and TNF-α (reported to go together with neuropathic pain (73, 74) (**Figure 5**), which mediate nerve injury indirectly. These data suggested that the effect of EA on metabolic disorder influenced the level of AGEs-RAGE and the inflammatory signals.

As the main enzymatic detoxification system of MG (a major precursor of AGEs that have been causally associated with the induction of neuropathic pain (75), the glyoxalase enzyme system, specifically glyoxalase I (GLO1), is responsible for detoxifying them by converting them to D-lactate, thus suppressing the formation of methylglyoxal-derived AGEs and providing primary defense against the reaction of associated glycation (76, 77). In hyperglycemia, the accumulation of MG damages the glyoxalase system, which in turn increases MG, forming a vicious cycle. The possible ways of GLO1 activation, such as nitric oxide (NO) inhibition, Nrf2 activation, and GSH synthesis, were reported to be regulated by EA treatment (78–81). The metabolism of MG mediated by the glyoxalase system depends on glutathione (GSH), which is a crucial co-enzyme of GLO1. Cellular GSH concentration is directly proportional to the *in situ* activity of GLO1 and is related to the antioxidant effect mediated by GSH (82, 83). It is proven that the antioxidant effect in the nerve system of EA is associated with the modulation of ROS and GSH (84). EA mediates the antioxidant effect through the upregulation of glutathione reductase (GR) and GSH, thus protecting the nerve system (85). According to the positive effect on AGEs reduction, we assumed that the effect of EA on metabolic regulation and antioxidation might have a positive effect on GLO1, and that was proven in this research with the significant increase of GLO1 and GSH after EA treatment (**Figure 6**). Since it is endogenously formed from MG through the glyoxalase system, D-lactate is a surrogate and qualitative indicator of MG flux and partly reflects the level of MG and the intensity of dicarbonyl. Besides, the increased level of D-lactate is most common in people with diabetes or obesity compared to others, indicating its importance in the evaluation of metabolic function (86–88). To further explore the regulation of the glyoxalase system, we detected the concentration of D-lactate (**Figures 6D–H**). The results suggest positive changes in the glyoxalase system.

In this study, we speculated that the reduction of AGEs was partly related to the activation of GLO1. Interestingly, the results showed a certain distance between AGE-RAGE and GLO1 expression in footpad skin. EA treatment almost completely improved AGEs and RAGE but partially ameliorated GLO1 in footpad skin. One explanation could be that EA might reduce AGEs and RAGE in other ways, not only by strengthening their detoxification. High-fat feed, especially containing animal fat, could lead to a high level of AGEs in circulation and tissues (89). AGEs and RAGE were reported to increase in hyperglycemia and hyperlipidemia (90), and that is the characteristic of the HFD-STZ-induced animal models. In this research, decreased food intake, reduced blood glucose, and improved dyslipidemia were found after EA treatment (**Figures 1C, F**), and that could exert an influence on AGEs accumulation.

**Figure 7:**
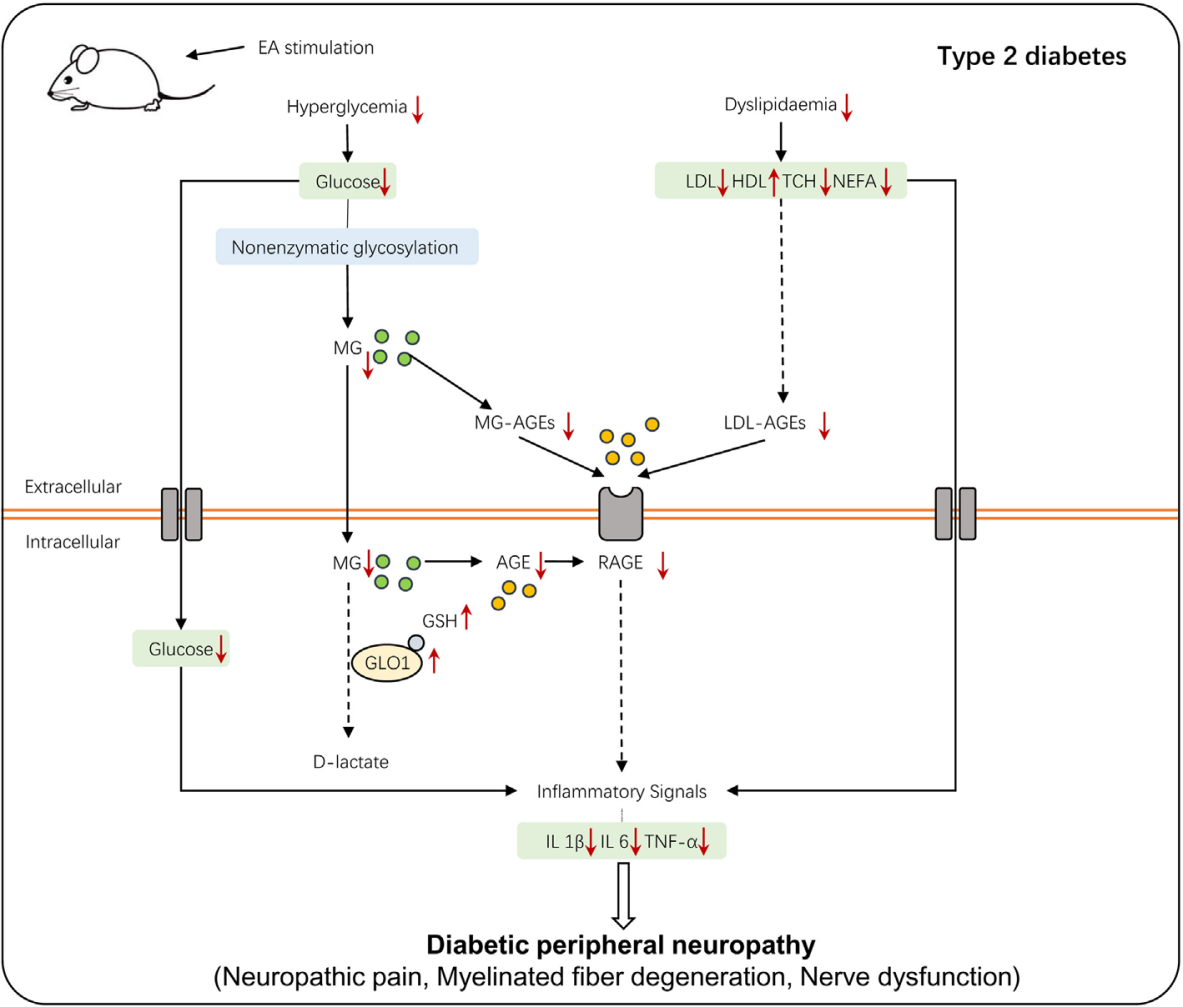
The schematic diagram of EA stimulation on T2DM-induced peripheral neuropathy. T2DM-induced hyperglycemia and dyslipidemia lead to the accumulation of AGEs and interaction of AGE-RAGE, which alters a series of inflammatory signals and eventually causes DPN. EA stimulation regulates glycolipid metabolism, which then activates the glyoxalase system and enhances MG detoxification, alleviating the hyperalgesia of DPN.

Rare studies report the role of EA in the process in which the human organism keeps the balance between hyperglycemia-induced metabolic faculty and the influence on DPN development based on it. To our knowledge, this is the first research establishing a connection between the positive effect conferred by EA and the regulation of the AGE/RAGE axis mediated by the glyoxalase system. Our research reported the underlying mechanism of the therapeutic treatment mediated by EA in diabetic neuropathic pain from the perspective of the its regulation on metabolism and the secondary influence on the GLO/AGE/RAGE axis (**Figure 7**), and it may provide a therapeutic strategy of T2DM-induced neuropathy.

There are several limitations in this research. First, the exact regulatory mechanism in the activation of GLO1 and its relationship with AGE-RAGE have not been clarified. Apart from the possible ways of GLO1 activation, such as nitric oxide (NO) inhibition, Nrf2 activation, and GSH synthesis, which were reported to be regulated by EA treatment (78–81), we infer that there is a link between metabolism regulation, especially glycolysis, and GLO1 activation, and EA treatment may play an essential role among them; whether the effect of EA treatment on GLO1 activation can be identified from metabolism regulation is unknown. Additionally, it is still controversial whether it is necessary to set up a sham-operated group in the research of EA, a sham-EA group was not applied in this research (91). Therefore the treatment of DPN may not entirely be mediated by EA in theory. since it is reported that gastric and intestinal electrical stimulation (GIES) decreases postprandial blood glucose levels and regulates metabolism in rats (92–94). In this research, though we set the pair of needles as close as possible, the spread of current and its possible stimulation on the small intestine was inevitable when 2 mA was applied, so the effect of EA on metabolism regulation may partially be associated with that. Further research will target the energy metabolism-related mechanism conferred by EA treatment and the optimal EA parameters. For a more precise description of the EA effect, we will include a sham group in the further study.

## Data Availability Statement

The raw data supporting the conclusions of this article will be made available by the authors, without undue reservation.

## Ethics Statement

The animal study was reviewed and approved by the Institutional Animal Care and Use Committee of Nanjing University of Chinese Medicine.

## Author Contributions

BX, ZY, and QL conceived and designed the experiments. XW performed the experiments and wrote the manuscript. XH and MG performed the experiments. XW, XH, and QL analyzed the data. All authors read and approved the final version of the article to be published.

## Funding

This work was supported by the National Natural Science Foundation of China (No. 81873238, No. 82074532, No. 81574071, and No. 81673883); the Leading Talents of Traditional Chinese Medicine in Jiangsu (SLJ0225); the Priority Academic Program Development of Jiangsu Higher Education Institutions (PAPD); the Open Projects of the Discipline of Chinese Medicine of Nanjing University of Chinese Medicine supported by the Subject of Academic Priority Discipline of Jiangsu Higher Education Institutions (No. ZYX03KF012); the Natural Science Foundation of Jiangsu Province (No. BK20181420); and the Postgraduate Research & Practice Innovation Program of Jiangsu Province (KYCX20_1539).

## Conflict of Interest

The authors declare that the research was conducted in the absence of any commercial or financial relationships that could be construed as a potential conflict of interest.

## References

[1] Saeedi P Petersohn I Salpea P Malanda B Karuranga S Unwin N. Global and Regional Diabetes Prevalence Estimates for 2019 and Projections for 2030 and 2045: Results From the International Diabetes Federation Diabetes Atlas, 9(th) Edition. Diabetes Res Clin Pract (2019) 157:107843. 10.1016/j.diabres.2019.107843 31518657

[2] Cho NH Shaw JE Karuranga S Huang Y da Rocha Fernandes JD Ohlrogge AW. IDF Diabetes Atlas: Global Estimates of Diabetes Prevalence for 2017 and Projections for 2045. Diabetes Res Clin Pract (2018) 138:271–81. 10.1016/j.diabres.2018.02.023 29496507

[3] Jack M Wright D. Role of Advanced Glycation Endproducts and Glyoxalase I in Diabetic Peripheral Sensory Neuropathy. Transl Res (2012) 159:355–65. 10.1016/j.trsl.2011.12.004 PMC3329218 22500508

[4] Abbott CA Malik RA van Ross ER Kulkarni J Boulton AJ. Prevalence and Characteristics of Painful Diabetic Neuropathy in a Large Community-Based Diabetic Population in the U.K. Diabetes Care (2011) 34:2220–4. 10.2337/dc11-1108 PMC3177727 21852677

[5] Shillo P Sloan G Greig M Hunt L Selvarajah D Elliott J. Painful and Painless Diabetic Neuropathies: What Is the Difference? Curr Diabetes Rep (2019) 19:32. 10.1007/s11892-019-1150-5 PMC6505492 31065863

[6] Tecilazich F Dinh T Lyons TE Guest J Villafuerte RA Sampanis C. Postexercise Phosphocreatine Recovery, An Index of Mitochondrial Oxidative Phosphorylation, is Reduced in Diabetic Patients With Lower Extremity Complications. J Vasc Surg (2013) 57:997–1005. 10.1016/j.jvs.2012.10.011 23465172 PMC3612142

[7] Yang J Zhao Z Yuan H Ma X Li Y Wang H. The Mechanisms of Glycemic Variability Accelerate Diabetic Central Neuropathy and Diabetic Peripheral Neuropathy in Diabetic Rats. Biochem Biophys Res Commun (2019) 510:35–41. 10.1016/j.bbrc.2018.12.179 30660367

[8] Moustafa PE Abdelkader NF El Awdan SA El-Shabrawy OA Zaki HF. Liraglutide Ameliorated Peripheral Neuropathy in Diabetic Rats: Involvement of Oxidative Stress, Inflammation and Extracellular Matrix Remodeling. J Neurochem (2018) 146:173–85. 10.1111/jnc.14336 29572844

[9] Sugimoto K Nishizawa Y Horiuchi S Yagihashi S. Localization in Human Diabetic Peripheral Nerve of N(epsilon)-Carboxymethyllysine-Protein Adducts, an Advanced Glycation Endproduct. Diabetologia (1997) 40:1380–7. 10.1007/s001250050839 9447944

[10] Lukic IK Humpert PM Nawroth PP Bierhaus A. The RAGE Pathway: Activation and Perpetuation in the Pathogenesis of Diabetic Neuropathy. Ann N Y Acad Sci (2008) 1126:76–80. 10.1196/annals.1433.059 18448798

[11] Huang CL Tsai PS Wang TY Yan LP Xu HZ Huang CJ. Acupuncture Stimulation of ST36 (Zusanli) Attenuates Acute Renal But Not Hepatic Injury in Lipopolysaccharide-Stimulated Rats. Anesth Analg (2007) 104:646–54. 10.1213/01.ane.0000255288.68199.eb 17312224

[12] Firouzjaei A Li GC Wang N Liu WX Zhu BM. Comparative Evaluation of the Therapeutic Effect of Metformin Monotherapy With Metformin and Acupuncture Combined Therapy on Weight Loss and Insulin Sensitivity in Diabetic Patients. Nutr Diabetes (2016) 6:e209. 10.1038/nutd.2016.16 27136447 PMC4895377

[13] Lan D Xu N Sun J Li Z Liao R Zhang H. Electroacupuncture Mitigates Endothelial Dysfunction *via* Effects on the PI3K/Akt Signalling Pathway in High Fat Diet-Induced Insulin-Resistant Rats. Acupunct Med (2018) 36:162–9. 10.1136/acupmed-2016-011253 29502072

[14] Bril V England J Franklin GM Backonja M Cohen J Del Toro D. Evidence-Based Guideline: Treatment of Painful Diabetic Neuropathy: Report of the American Academy of Neurology, the American Association of Neuromuscular and Electrodiagnostic Medicine, and the American Academy of Physical Medicine and Rehabilitation. Neurology (2011) 76:1758–65. 10.1212/WNL.0b013e3182166ebe PMC3100130 21482920

[15] Ulloa L Quiroz-Gonzalez S Torres-Rosas R. Nerve Stimulation: Immunomodulation and Control of Inflammation. Trends Mol Med (2017) 23:1103–20. 10.1016/j.molmed.2017.10.006 PMC5724790 29162418

[16] Xu YD Cui JM Wang Y Yin LM Gao CK Liu XY. Proteomic Analysis Reveals the Deregulation of Inflammation-Related Proteins in Acupuncture-Treated Rats With Asthma Onset. Evid Based Complement Alternat Med (2012) 2012:850512. 10.1155/2012/850512 23304218 PMC3523810

[17] Liao HY Hsieh CL Huang CP Lin YW. Electroacupuncture Attenuates CFA-Induced Inflammatory Pain by Suppressing Nav1.8 Through S100B, TRPV1, Opioid, and Adenosine Pathways in Mice. Sci Rep (2017) 7:42531. 10.1038/srep42531 28211895 PMC5304170

[18] Shin KM Lee S Lee EY Kim CH Kang JW Lee CK. Electroacupuncture for Painful Diabetic Peripheral Neuropathy: A Multicenter, Randomized, Assessor-Blinded, Controlled Trial. Diabetes Care (2018) 41:e141–2. 10.2337/dc18-1254 30061320

[19] Fei X He X Tai Z Wang H Qu S Chen L. Electroacupuncture Alleviates Diabetic Neuropathic Pain in Rats by Suppressing P2X3 Receptor Expression in Dorsal Root Ganglia. Purinergic Signal (2020) 16(4):491–502. 10.1007/s11302-020-09728-9 33011961 PMC7855163

[20] Chen IJ Yeh YH Hsu CH. Therapeutic Effect of Acupoint Catgut Embedding in Abdominally Obese Women: A Randomized, Double-Blind, Placebo-Controlled Study. J Womens Health (Larchmt) (2018) 27:782–90. 10.1089/jwh.2017.6542 29723106

[21] Zhu HD Gong Z Hu BW Wei QL Kong J Peng CB. The Efficacy and Safety of Transcutaneous Acupoint Interferential Current Stimulation for Cancer Pain Patients With Opioid-Induced Constipation: A Prospective Randomized Controlled Study. Integr Cancer Ther (2018) 17:437–43. 10.1177/1534735417734910 PMC6041914 29076387

[22] Liu S Wang ZF Su YS Ray RS Jing XH Wang YQ. Somatotopic Organization and Intensity Dependence in Driving Distinct NPY-Expressing Sympathetic Pathways by Electroacupuncture. Neuron (2020) 108:436–450 e7. 10.1016/j.neuron.2020.07.015 32791039 PMC7666081

[23] Yu Z Xia Y Ju C Shao Q Mao Z Gu Y. Electroacupuncture Regulates Glucose-Inhibited Neurons in Treatment of Simple Obesity. Neural Regener Res (2013) 8:809–16. 10.3969/j.issn.1673-5374.2013.09.005 PMC4146081 25206728

[24] Tang Q Lu M Xu B Wang Y Lu S Yu Z. Electroacupuncture Regulates Inguinal White Adipose Tissue Browning by Promoting Sirtuin-1-Dependent PPARgamma Deacetylation and Mitochondrial Biogenesis. Front Endocrinol (Lausanne) (2020) 11:607113. 10.3389/fendo.2020.607113 33551999 PMC7859442

[25] Lu M He Y Gong M Li Q Tang Q Wang X. Role of Neuro-Immune Cross-Talk in the Anti-Obesity Effect of Electro-Acupuncture. Front Neurosci (2020) 14:151. 10.3389/fnins.2020.00151 32180699 PMC7059539

[26] Zhu X Liu Z Qin Y Niu W Wang Q Li L. Analgesic Effects of Electroacupuncture at ST25 and CV12 in a Rat Model of Postinflammatory Irritable Bowel Syndrome Visceral Pain. Acupunct Med (2018) 36:240–6. 10.1136/acupmed-2016-011320 29720377

[27] Li ZY Huang Y Yang YT Zhang D Zhao Y Hong J. Moxibustion Eases Chronic Inflammatory Visceral Pain Through Regulating MEK, ERK and CREB in Rats. World J Gastroenterol (2017) 23:6220–30. 10.3748/wjg.v23.i34.6220 PMC5603488 28974888

[28] Kim K Lee S. Intradermal Acupuncture Along With Analgesics for Pain Control in Advanced Cancer Cases: A Pilot, Randomized, Patient-Assessor-Blinded, Controlled Trial. Integr Cancer Ther (2018) 17:1137–43. 10.1177/1534735418786797 PMC6247560 30009652

[29] Srinivasan K Viswanad B Asrat L Kaul CL Ramarao P. Combination of High-Fat Diet-Fed and Low-Dose Streptozotocin-Treated Rat: A Model for Type 2 Diabetes and Pharmacological Screening. Pharmacol Res (2005) 52:313–20. 10.1016/j.phrs.2005.05.004 15979893

[30] Reed MJ Meszaros K Entes LJ Claypool MD Pinkett JG Gadbois TM. A New Rat Model of Type 2 Diabetes: The Fat-Fed, Streptozotocin-Treated Rat. Metabolism (2000) 49:1390–4. 10.1053/meta.2000.17721 11092499

[31] Gunnarsson R Berne C Hellerstrom C. Cytotoxic Effects of Streptozotocin and N-Nitrosomethylurea on the Pancreatic B Cells With Special Regard to the Role of Nicotinamide-Adenine Dinucleotide. Biochem J (1974) 140:487–94. 10.1042/bj1400487 PMC1168026 4374939

[32] Agarwal N Helmstadter J Rojas DR Bali KK Gangadharan V Kuner R. Evoked Hypoalgesia is Accompanied by Tonic Pain and Immune Cell Infiltration in the Dorsal Root Ganglia at Late Stages of Diabetic Neuropathy in Mice. Mol Pain (2018) 14:1744806918817975. 10.1177/1744806918817975 30453826 PMC6311571

[33] O’Brien PD Hinder LM Rumora AE Hayes JM Dauch JR Backus C. Juvenile Murine Models of Prediabetes and Type 2 Diabetes Develop Neuropathy. Dis Model Mech (2018) 11(12):dmm037374. 10.1242/dmm.037374 30446513 PMC6307897

[34] Adki KM Kulkarni YA. Neuroprotective Effect of Paeonol in Streptozotocin-Induced Diabetes in Rats. Life Sci (2021) 271:119202. 10.1016/j.lfs.2021.119202 33577853

[35] Yerra VG Kalvala AK Sherkhane B Areti A Kumar A. Adenosine Monophosphate-Activated Protein Kinase Modulation by Berberine Attenuates Mitochondrial Deficits and Redox Imbalance in Experimental Diabetic Neuropathy. Neuropharmacology (2018) 131:256–70. 10.1016/j.neuropharm.2017.12.029 29273519

[36] Ali S Driscoll HE Newton VL Gardiner NJ. Matrix Metalloproteinase-2 is Downregulated in Sciatic Nerve by Streptozotocin Induced Diabetes and/or Treatment With Minocycline: Implications for Nerve Regeneration. Exp Neurol (2014) 261:654–65. 10.1016/j.expneurol.2014.08.017 PMC4199570 25158309

[37] Chandrasekaran K Salimian M Konduru SR Choi J Kumar P Long A. Overexpression of Sirtuin 1 Protein in Neurons Prevents and Reverses Experimental Diabetic Neuropathy. Brain (2019) 142:3737–52. 10.1093/brain/awz324 PMC6885680 31754701

[38] Rahman MH Jha MK Kim JH Nam Y Lee MG Go Y. Pyruvate Dehydrogenase Kinase-Mediated Glycolytic Metabolic Shift in the Dorsal Root Ganglion Drives Painful Diabetic Neuropathy. J Biol Chem (2016) 291:6011–25. 10.1074/jbc.M115.699215 PMC4786733 26769971

[39] Obrosova IG Ilnytska O Lyzogubov VV Pavlov IA Mashtalir N Nadler JL. High-Fat Diet Induced Neuropathy of Pre-Diabetes and Obesity: Effects of “Healthy” Diet and Aldose Reductase Inhibition. Diabetes (2007) 56:2598–608. 10.2337/db06-1176 17626889

[40] Boyle JP Thompson TJ Gregg EW Barker LE Williamson DF. Projection of the Year 2050 Burden of Diabetes in the US Adult Population: Dynamic Modeling of Incidence, Mortality, and Prediabetes Prevalence. Popul Health Metr (2010) 8:29. 10.1186/1478-7954-8-29 20969750 PMC2984379

[41] Tesfaye S Boulton AJ Dickenson AH. Mechanisms and Management of Diabetic Painful Distal Symmetrical Polyneuropathy. Diabetes Care (2013) 36:2456–65. 10.2337/dc12-1964 PMC3747929 23970715

[42] Davidson EP Coppey LJ Shevalye H Obrosov A Yorek MA. Effect of Dietary Content of Menhaden Oil With or Without Salsalate on Neuropathic Endpoints in High-Fat-Fed/Low-Dose Streptozotocin-Treated Sprague Dawley Rats. J Diabetes Res (2018) 2018:2967127. 10.1155/2018/2967127 30057911 PMC6051246

[43] Skovso S. Modeling Type 2 Diabetes in Rats Using High Fat Diet and Streptozotocin. J Diabetes Investig (2014) 5:349–58. 10.1111/jdi.12235 PMC4210077 25411593

[44] Magalhaes DA Kume WT Correia FS Queiroz TS Allebrandt Neto EW Santos MPD. High-Fat Diet and Streptozotocin in the Induction of Type 2 Diabetes Mellitus: A New Proposal. Acad Bras Cienc (2019) 91:e20180314. 10.1590/0001-3765201920180314 30916157

[45] Heydemann A. An Overview of Murine High Fat Diet as a Model for Type 2 Diabetes Mellitus. J Diabetes Res (2016) 2016:2902351. 10.1155/2016/2902351 27547764 PMC4983380

[46] Vornoli A Pozzo L Della Croce CM Gervasi PG Longo V. Drug Metabolism Enzymes in a Steatotic Model of Rat Treated With a High Fat Diet and a Low Dose of Streptozotocin. Food Chem Toxicol (2014) 70:54–60. 10.1016/j.fct.2014.04.042 24815820

[47] Cancello R Clement K. Is Obesity an Inflammatory Illness? Role of Low-Grade Inflammation and Macrophage Infiltration in Human White Adipose Tissue. BJOG (2006) 113:1141–7. 10.1111/j.1471-0528.2006.01004.x 16903845

[48] Feng Y Fang Y Wang Y Hao Y. Acupoint Therapy on Diabetes Mellitus and Its Common Chronic Complications: A Review of Its Mechanisms. BioMed Res Int (2018) 2018:3128378. 10.1155/2018/3128378 30426006 PMC6217896

[49] Ueda H. Peripheral Mechanisms of Neuropathic Pain - Involvement of Lysophosphatidic Acid Receptor-Mediated Demyelination. Mol Pain (2008) 4:11. 10.1186/1744-8069-4-11 18377664 PMC2365930

[50] Feldman EL Nave KA Jensen TS Bennett DLH. New Horizons in Diabetic Neuropathy: Mechanisms, Bioenergetics, and Pain. Neuron (2017) 93:1296–313. 10.1016/j.neuron.2017.02.005 PMC5400015 28334605

[51] Modrak M Talukder MAH Gurgenashvili K Noble M Elfar JC. Peripheral Nerve Injury and Myelination: Potential Therapeutic Strategies. J Neurosci Res (2020) 98:780–95. 10.1002/jnr.24538 PMC7072007 31608497

[52] Brushart TM Jari R Verge V Rohde C Gordon T. Electrical Stimulation Restores the Specificity of Sensory Axon Regeneration. Exp Neurol (2005) 194:221–9. 10.1016/j.expneurol.2005.02.007 15899259

[53] Al-Majed AA Neumann CM Brushart TM Gordon T. Brief Electrical Stimulation Promotes the Speed and Accuracy of Motor Axonal Regeneration. J Neurosci (2000) 20:2602–8. 10.1523/JNEUROSCI.20-07-02602.2000 PMC6772244 10729340

[54] Liu LA Wang ZQ Fu JJ Du QQ Zhang YY Jiao CC. Comparative Observation on Electroacupuncture and Manual Acupuncture in Rabbits With Facial Nerve Injury by Electron Microscope. Zhen Ci Yan Jiu (2017) 42:423–8. 29105471

[55] Ding Y Zhang RY He B Liu Z Zhang K Ruan JW. Combination of Electroacupuncture and Grafted Mesenchymal Stem Cells Overexpressing TrkC Improves Remyelination and Function in Demyelinated Spinal Cord of Rats. Sci Rep (2015) 5:9133. 10.1038/srep09133 25779025 PMC5390924

[56] Young MJ Veves A Smith JV Walker MG Boulton AJ. Restoring Lower Limb Blood Flow Improves Conduction Velocity in Diabetic Patients. Diabetologia (1995) 38:1051–4. 10.1007/BF00402174 8591818

[57] Liampas A Rekatsina M Vadalouca A Paladini A Varrassi G Zis P. Pharmacological Management of Painful Peripheral Neuropathies: A Systematic Review. Pain Ther (2020) 37(10):4096–106. 10.1007/s40122-020-00210-3 32809209

[58] Misur I Zarkovic K Barada A Batelja L Milicevic Z Turk Z. Advanced Glycation Endproducts in Peripheral Nerve in Type 2 Diabetes With Neuropathy. Acta Diabetol (2004) 41:158–66. 10.1007/s00592-004-0160-0 15660198

[59] Pop-Busui R Sima A Stevens M. Diabetic Neuropathy and Oxidative Stress. Diabetes Metab Res Rev (2006) 22:257–73. 10.1002/dmrr.625 16506271

[60] Tian D Mo F Cai X Miao Z Xiao F Chang Y. Acupuncture Relieves Motion Sickness *via* the IRbeta-ERK1/2-Dependent Insulin Receptor Signalling Pathway. Acupunct Med (2018) 36:153–61. 10.1136/acupmed-2016-011202 29436382

[61] Liang F Koya D. Acupuncture: Is It Effective for Treatment of Insulin Resistance? Diabetes Obes Metab (2010) 12:555–69. 10.1111/j.1463-1326.2009.01192.x 20590731

[62] Hu H. A Review of Treatment of Diabetes by Acupuncture During the Past Forty Years. J Tradit Chin Med (1995) 15:145–54. 7650966

[63] Li Y Qian ZY Cheng K Zhao L Shen XY Deng HP. Effect of Compound Laser Acupuncture-Moxibustion on Blood Glucose, Fasting Insulin and Blood Lipids Levels in Type 2 Diabetic Rats. Chin J Integr Med (2020) 26:33–8. 10.1007/s11655-019-3084-9 31776963

[64] Lee JD Jang MH Kim EH Kim CJ. Acupuncture Decreases Neuropeptide Y Expression in the Hypothalamus of Rats With Streptozotocin-Induced Diabetes. Acupunct Electrother Res (2004) 29:73–82. 10.3727/036012904815901533 15382790

[65] Vincent AM Perrone L Sullivan KA Backus C Sastry AM Lastoskie C. Receptor for Advanced Glycation End Products Activation Injures Primary Sensory Neurons *via* Oxidative Stress. Endocrinology (2007) 148:548–58. 10.1210/en.2006-0073 17095586

[66] Zochodne DW. Mechanisms of Diabetic Neuron Damage: Molecular Pathways. Handb Clin Neurol (2014) 126:379–99. 10.1016/B978-0-444-53480-4.00028-X 25410235

[67] Lander HM Tauras JM Ogiste JS Hori O Moss RA Schmidt AM. Activation of the Receptor for Advanced Glycation End Products Triggers a P21(Ras)-Dependent Mitogen-Activated Protein Kinase Pathway Regulated by Oxidant Stress. J Biol Chem (1997) 272:17810–4. 10.1074/jbc.272.28.17810 9211935

[68] Bierhaus A Humpert PM Morcos M Wendt T Chavakis T Arnold B. The Receptor for Advanced Glycation End Products. J Mol Med (Berl) (2005) 83:876–86. 10.1007/s00109-005-0688-7 16133426

[69] Chang SL Lin JG Chi TC Liu IM Cheng JT. An Insulin-Dependent Hypoglycaemia Induced by Electroacupuncture at the Zhongwan (CV12) Acupoint in Diabetic Rats. Diabetologia (1999) 42:250–5. 10.1007/s001250051146 10064107

[70] Su TF Zhang LH Peng M Wu CH Pan W Tian B. Cannabinoid CB2 Receptors Contribute to Upregulation of Beta-Endorphin in Inflamed Skin Tissues by Electroacupuncture. Mol Pain (2011) 7:98. 10.1186/1744-8069-7-98 22177137 PMC3281798

[71] Mousa SA Shaqura M Brendl U Al-Khrasani M Furst S Schafer M. Involvement of the Peripheral Sensory and Sympathetic Nervous System in the Vascular Endothelial Expression of ICAM-1 and the Recruitment of Opioid-Containing Immune Cells to Inhibit Inflammatory Pain. Brain Behav Immun (2010) 24:1310–23. 10.1016/j.bbi.2010.06.008 20600813

[72] Johnston GR Webster NR. Cytokines and the Immunomodulatory Function of the Vagus Nerve. Br J Anaesth (2009) 102:453–62. 10.1093/bja/aep037 19258380

[73] Uceyler N Riediger N Kafke W Sommer C. Differential Gene Expression of Cytokines and Neurotrophic Factors in Nerve and Skin of Patients With Peripheral Neuropathies. J Neurol (2015) 262:203–12. 10.1007/s00415-014-7556-8 25371017

[74] Kellogg AP Wiggin TD Larkin DD Hayes JM Stevens MJ Pop-Busui R. Protective Effects of Cyclooxygenase-2 Gene Inactivation Against Peripheral Nerve Dysfunction and Intraepidermal Nerve Fiber Loss in Experimental Diabetes. Diabetes (2007) 56:2997–3005. 10.2337/db07-0740 17720896

[75] Huang Q Chen Y Gong N Wang YX. Methylglyoxal Mediates Streptozotocin-Induced Diabetic Neuropathic Pain *via* Activation of the Peripheral TRPA1 and Nav1.8 Channels. Metabolism (2016) 65:463–74. 10.1016/j.metabol.2015.12.002 26975538

[76] Rabbani N Thornalley PJ. Glyoxalase in Diabetes, Obesity and Related Disorders. Semin Cell Dev Biol (2011) 22:309–17. 10.1016/j.semcdb.2011.02.015 21335095

[77] Thornalley PJ. Glyoxalase I–structure, Function and a Critical Role in the Enzymatic Defence Against Glycation. Biochem Soc Trans (2003) 31:1343–8. 10.1042/bst0311343 14641060

[78] He Y Zhou C Huang M Tang C Liu X Yue Y. Glyoxalase System: A Systematic Review of its Biological Activity, Related-Diseases, Screening Methods and Small Molecule Regulators. BioMed Pharmacother (2020) 131:110663. 10.1016/j.biopha.2020.110663 32858501

[79] Maher P Dargusch R Ehren JL Okada S Sharma K Schubert D. Fisetin Lowers Methylglyoxal Dependent Protein Glycation and Limits the Complications of Diabetes. PloS One (2011) 6:e21226. 10.1371/journal.pone.0021226 21738623 PMC3124487

[80] Cheng AS Cheng YH Chiou CH Chang TL. Resveratrol Upregulates Nrf2 Expression to Attenuate Methylglyoxal-Induced Insulin Resistance in Hep G2 Cells. J Agric Food Chem (2012) 60:9180–7. 10.1021/jf302831d 22917016

[81] Miller AG Smith DG Bhat M Nagaraj RH. Glyoxalase I is Critical for Human Retinal Capillary Pericyte Survival Under Hyperglycemic Conditions. J Biol Chem (2006) 281:11864–71. 10.1074/jbc.M513813200 16505483

[82] Abordo EA Minhas HS Thornalley PJ. Accumulation of Alpha-Oxoaldehydes During Oxidative Stress: A Role in Cytotoxicity. Biochem Pharmacol (1999) 58:641–8. 10.1016/S0006-2952(99)00132-X 10413301

[83] Rabbani N Xue M Thornalley PJ. Dicarbonyls and Glyoxalase in Disease Mechanisms and Clinical Therapeutics. Glycoconj J (2016) 33:513–25. 10.1007/s10719-016-9705-z PMC4975768 27406712

[84] Erthal V Maria-Ferreira D Werner MF Baggio CH Nohama P. Anti-Inflammatory Effect of Laser Acupuncture in ST36 (Zusanli) Acupoint in Mouse Paw Edema. Lasers Med Sci (2016) 31:315–22. 10.1007/s10103-015-1845-z 26738499

[85] Du SQ Wang XR Zhu W Ye Y Yang JW Ma SM. Acupuncture Inhibits TXNIP-Associated Oxidative Stress and Inflammation to Attenuate Cognitive Impairment in Vascular Dementia Rats. CNS Neurosci Ther (2018) 24:39–46. 10.1111/cns.12773 29110407 PMC6489958

[86] Masania J Malczewska-Malec M Razny U Goralska J Zdzienicka A Kiec-Wilk B. Dicarbonyl Stress in Clinical Obesity. Glycoconj J (2016) 33:581–9. 10.1007/s10719-016-9692-0 PMC4975769 27338619

[87] Scheijen JL Hanssen NM van de Waarenburg MP Jonkers DM Stehouwer CD Schalkwijk CG. L(+) and D(-) Lactate Are Increased in Plasma and Urine Samples of Type 2 Diabetes as Measured by a Simultaneous Quantification of L(+) and D(-) Lactate by Reversed-Phase Liquid Chromatography Tandem Mass Spectrometry. Exp Diabetes Res (2012) 2012:234812. 10.1155/2012/234812 22474418 PMC3310144

[88] Talasniemi JP Pennanen S Savolainen H Niskanen L Liesivuori J. Analytical Investigation: Assay of D-Lactate in Diabetic Plasma and Urine. Clin Biochem (2008) 41:1099–103. 10.1016/j.clinbiochem.2008.06.011 18638467

[89] Uribarri J Woodruff S Goodman S Cai W Chen X Pyzik R. Advanced Glycation End Products in Foods and a Practical Guide to Their Reduction in the Diet. J Am Diet Assoc (2010) 110:911–16 e12. 10.1016/j.jada.2010.03.018 20497781 PMC3704564

[90] Gaens KH Stehouwer CD Schalkwijk CG. Advanced Glycation Endproducts and Its Receptor for Advanced Glycation Endproducts in Obesity. Curr Opin Lipidol (2013) 24:4–11. 10.1097/MOL.0b013e32835aea13 23298958

[91] Langevin HM Wayne PM Macpherson H Schnyer R Milley RM Napadow V. Paradoxes in Acupuncture Research: Strategies for Moving Forward. Evid Based Complement Alternat Med (2011) 2011:180805. 10.1155/2011/180805 20976074 PMC2957136

[92] Khawaled R Blumen G Fabricant G Ben-Arie J Shikora S. Intestinal Electrical Stimulation Decreases Postprandial Blood Glucose Levels in Rats. Surg Obes Relat Dis (2009) 5:692–7. 10.1016/j.soard.2009.05.013 19640804

[93] Yin J Chen JD. Mechanisms and Potential Applications of Intestinal Electrical Stimulation. Dig Dis Sci (2010) 55:1208–20. 10.1007/s10620-009-0884-3 19629689

[94] Ye F Liu Y Li S Chen JDZ. Hypoglycemic Effects of Intestinal Electrical Stimulation by Enhancing Nutrient-Stimulated Secretion of GLP-1 in Rats. Obes Surg (2018) 28:2829–35. 10.1007/s11695-018-3257-1 29728986

